# Global burden, subtype, risk factors and etiological analysis of enteric infections from 1990-2021: population based study

**DOI:** 10.3389/fcimb.2025.1527765

**Published:** 2025-03-20

**Authors:** Youao Zhang, Yuran Zhang, Zhifeng Chen, Zixuan Jia, Yulan Yu, Jieyan Wang, Hui Liang

**Affiliations:** ^1^ Department of Urology, People’s Hospital of Longhua, Shenzhen, Guangdong, China; ^2^ Nanfang Hospital, The First Clinical Medical College of Southern Medical University, Guangzhou, Guangdong, China; ^3^ School of Chinese Medicine, Southern Medical University, Guangzhou, China

**Keywords:** enteric infections, global burden of disease, epidemiology, trend analysis, risk factors, etiological analysis

## Abstract

**Background:**

Enteric infections represent a prevalent global health issue and contribute significantly to the global disease burden. This study aims to investigate the patterns and trends of enteric infections from 1990 to 2021, providing valuable insights for health policy formulation, medical resource allocation, and the optimization of patient management plans.

**Methods:**

We analyzed the Global Burden of Disease (GBD) 2021 for 21 regions and 204 countries to understand better the health burden using prevalence, incidence, mortality, and disability-adjusted life years (DALYs), subtype, risk factors, and etiology. We tested correlations with the Socio-demographic Index (SDI), and using decomposition analysis to dissect the reasons behind changes in epidemiological indicators of the disease.

**Results:**

In 2021, the age-standardized rates of prevalence, incidence, deaths, and DALYs per 100,000 population for enteric infections were 879.58, 577.21, 17.83, and 1020.15, respectively. Compared to 1990, these rates exhibited -0.18, -0.12, -0.73, and -0.72 changes. Gender and age analyses revealed a higher burden among females, those under 15 years old, and the elderly. Regions with low SDI had higher epidemiological indicators. The burden of Typhoid fever declines in high-development regions. Unsafe water sources were identified as the primary risk factor globally in both 1990 and 2021. Rotavirus was the leading cause of deaths and DALYs.

**Conclusion:**

This study highlights the complex epidemiological landscape of enteric infections, revealing variations in burden, risk factors, and etiological characteristics across age, gender, and geographical regions. It underscores the urgent need for healthcare professionals and policymakers to develop innovative prevention and healthcare strategies based on the current and evolving burden of enteric infections, to alleviate the global disease burden.

## Graphical Abstract

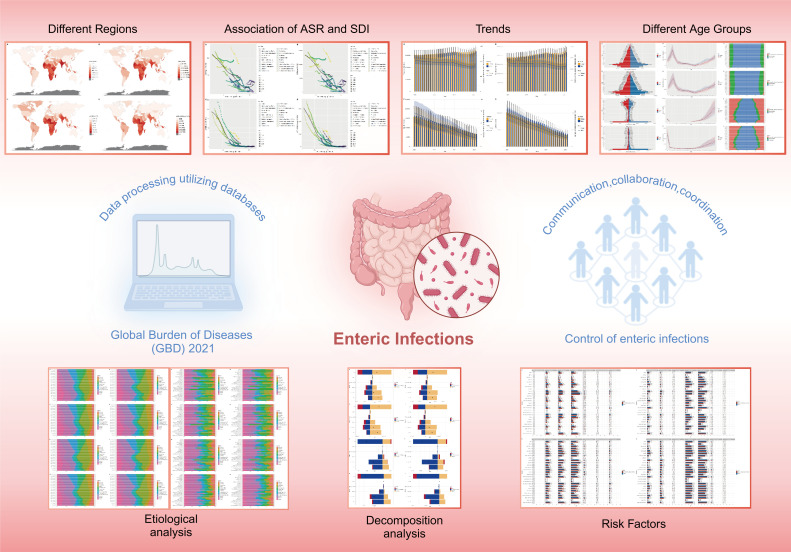

## Introduction

Enteric infections, as a category of diseases significantly affecting global public health, encompass gastrointestinal inflammation caused by various pathogens (Torgerson et al., 2015). These infections not only lead to a decrease in patients’ quality of life and increasing the risk of death (Hoivik et al., 2012), but also impose a significant economic burden at the social and family levels (Boccuzzi, 2003). Diarrheal diseases, a common symptom of gastrointestinal infections caused by diverse pathogens, pose a significant risk of dehydration and are the seventh leading cause of death in low-income countries according to the 2021 WHO statistics (World Health Organization, 2021). Typhoid fever, transmitted via contaminated food or water, can lead to severe fever, fatigue, and mortality. In low-income and middle-income countries, the average total cost of inpatient treatment per person ranges from $201 to $976 (Debellut et al., 2024). Paratyphoid A and B have clinical presentations similar to typhoid but are milder and shorter in duration. Invasive non-typhoidal Salmonella (iNTS) is a major health problem in low-income and underdeveloped countries in Africa and Asia that lack proper sanitation facilities (Hajra et al., 2023).

Therefore, the purpose of this analysis is to characterize the global burden of enteric infections and subtype in 21 regions and 204 countries and territories worldwide. The 2021 GBD conducted an in-depth assessment of the health hazards, with the support of more than 11,500 collaborators from 164 countries (GBD 2021 Causes of Death Collaborators, 2024; GBD 2021 Diseases and Injuries Collaborators, 2024; Sun et al., 2024; Tuo et al., 2024; Yang et al., 2024). Therefore, the GBD study is a reliable tool for understanding and assessing the current burden of enteric infections.

## Methods

### Data source

The present study extracted global incidence, prevalence, mortality, and DALYs (one DALY represents the loss of one year of full health due to premature death or disability), along with their 95% uncertainty intervals (UI), spanning from 1990 to 2021 from the GBD database (https://vizhub.healthdata.org/gbd-results/). Data on sex, age groups, population, and SDI were also included for comprehensive analysis.

### SDI analysis

The SDI was employed as a metric to assess the socio-economic status of countries, with a higher score indicating more robust socio-economic development. Regions were stratified into five quintiles based on the GBD studies: High SDI (>0.81), High-middle SDI (0.70–0.81), Middle SDI (0.61–0.69), Low-middle SDI (0.46–0.60), and Low SDI (<0.46) (GBD 2021 Causes of Death Collaborators, 2024; GBD 2021 Diseases and Injuries Collaborators, 2024). To examine the correlation between SDI and the burden of enteric infections, locally estimated scatterplot smoothing (loess) models were applied, utilizing the geom_smooth function within the ggplot2 package.

### Decomposition analysis

To quantify the driving factors behind the changes in the number of enteric infections, we estimated the relative contributions of aging, population growth, and epidemiological shifts. For this decomposition analysis, we employed the classic method developed by Das Gupta (1978), which utilizes algebraic decomposition techniques to break down the overall changes into the standardized impacts of each factor, thereby identifying the contribution of each component.

### Statistics analysis

Age-standardized prevalence rates (ASPR), age-standardized incidence rates (ASIR), age-standardized mortality rates (ASMR), and ASR of DALYs were expressed as predicted values per 100,000 population, including their 95% UI. All analytical procedures and graphical representations were executed using the statistical software R (version 4.4.1).

## Results

### Global level

#### Enteric infections

In 2021, the global prevalence was 67,826,600, with an ASPR of 879.58. Between 1990 and 2021, the ASPR decreased by 0.18, and the ASIR decreased by 0.12. The number of incident cases was 4,448,407,870 with an ASIR of 57,721.08. From 1990 to 2021, the number of deaths was 1,336,220 with an ASMR of 17.83; the ASMR decreased by 0.73 over this period. The global DALYs totaled 71,929,008 with an ASR of 1,020.15, and the ASR of DALYs decreased by 0.72 from 1990 to 2021 (**Table 1**).

**Table 1:** Prevalent cases, age-standardized prevalence rates, incident cases, age-standardized incidence rates, death cases, age-standardized mortality rates, DALYs, and age-standardized rates of DALYs for enteric infections in 1990 and 2021 for both sexes and rate change of age-standardized rates by GBD.

		1990		2021		1990–2021
		All-ages cases	Age-standardized rates per 100,000	All-ages cases	Age-standardized rates per 100,000	Rate change in age-standardized rates
		N (95%UI)		N (95%UI)		N (95%UI)
Global	Prevalence	61055404 (54000541 to 68729603)	1043.56 (925.18 to 1171.6)	67826600 (61771775 to 74750450)	879.58 (801.36 to 972.25)	-0.18 (-0.22 to -0.13)
	Incidence	3758659175 (3260094453 to 4255859151)	65466.27 (56954.84 to 73982.41)	4448407870 (3965332197 to 4991900224)	57721.08 (51210.03 to 64765.61)	-0.12 (-0.18 to -0.06)
	Deaths	3214610 (2574183 to 4002667)	60.58 (46.34 to 79.9)	1336220 (978118 to 1797317)	17.83 (13.38 to 23.5)	-0.73 (-0.77 to -0.68)
	DALYs	214438908 (173838488 to 252010744)	3337.13 (2684.15 to 3994.3)	71929008 (58296705 to 88193657)	1020.15 (822.7 to 1259.39)	-0.72 (-0.77 to -0.67)
Socio-demographic Index (SDI)						
High SDI	Prevalence	2551657 (2142234 to 3028519)	326.97 (269.31 to 394.92)	2767787 (2453972 to 3109622)	278.39 (235.41 to 326.08)	-0.15 (-0.19 to -0.11)
	Incidence	161510412 (133525808 to 191309059)	20689.81 (16749.3 to 24983.77)	173794217 (150709751 to 197313883)	17498.72 (14477.63 to 20576.96)	-0.15 (-0.19 to -0.11)
	Deaths	8064 (7098 to 9151)	0.84 (0.73 to 0.97)	27117 (22704 to 29833)	1.16 (1 to 1.26)	0.25 (0.07 to 0.41)
	DALYs	609189 (494654 to 742900)	77.57 (61.87 to 95.79)	731654 (631981 to 856337)	57.86 (47.68 to 71.48)	-0.3 (-0.39 to -0.23)
High-middle SDI	Prevalence	4085310 (3466586 to 4816257)	410.07 (344.78 to 486.18)	2745044 (2388543 to 3140651)	247.23 (209.74 to 289.69)	-0.4 (-0.44 to -0.36)
	Incidence	256269268 (212672970 to 302880831)	25845.86 (21357.63 to 30597.41)	180727882 (154454773 to 209294829)	16245.72 (13536.94 to 19130.51)	-0.37 (-0.41 to -0.33)
	Deaths	51860 (40808 to 62563)	5.26 (4.09 to 6.33)	18631 (13740 to 23200)	1.3 (1.01 to 1.59)	-0.77 (-0.81 to -0.74)
	DALYs	4232752 (3520463 to 4982910)	420.6 (349.94 to 498.14)	877647 (720773 to 1069220)	84.66 (70 to 102.51)	-0.82 (-0.84 to -0.78)
Middle SDI	Prevalence	18100901 (15818203 to 20694988)	970.33 (857.36 to 1097.96)	16130490 (14571235 to 18003830)	684.38 (614.99 to 767.47)	-0.31 (-0.34 to -0.26)
	Incidence	1116840410 (955571259 to 1281310554)	60646.64 (52576.63 to 68929.37)	1068928569 (943201109 to 1209397348)	45293.11 (39773.15 to 51308.45)	-0.26 (-0.3 to -0.21)
	Deaths	558050 (424745 to 706972)	41.96 (28.5 to 58.22)	188350 (121962 to 266788)	8.76 (5.79 to 12.13)	-0.8 (-0.84 to -0.76)
	DALYs	38118303 (30474442 to 45305880)	1972.46 (1539.6 to 2372.74)	8890401 (6992304 to 11176121)	426.35 (340.12 to 529.48)	-0.8 (-0.83 to -0.77)
Low-middle SDI	Prevalence	23906860 (21237278 to 26668660)	1752.18 (1587.72 to 1935.52)	26447136 (24067904 to 29066200)	1391.11 (1273.51 to 1522.18)	-0.23 (-0.27 to -0.19)
	Incidence	1469161007 (1275921726 to 1651651578)	110887.41 (98232.61 to 124029.01)	1735881923 (1544016836 to 1948698545)	91250.49 (81752.09 to 102132.36)	-0.19 (-0.23 to -0.14)
	Deaths	1623455 (1308956 to 2067629)	185.39 (136.59 to 261.93)	554032 (388724 to 819133)	42.1 (27.89 to 63.93)	-0.78 (-0.83 to -0.72)
	DALYs	102147738 (85140005 to 120396465)	6982.21 (5654.75 to 8895.33)	25272138 (20143943 to 32252792)	1482.56 (1168.18 to 1949.54)	-0.81 (-0.84 to -0.77)
Low SDI	Prevalence	12379983 (11164337 to 13664446)	2018.19 (1851.61 to 2198.63)	19703744 (18022741 to 21450172)	1774.15 (1648.72 to 1906.17)	-0.14 (-0.18 to -0.1)
	Incidence	752987003 (662421461 to 845942634)	125371.61 (112564.78 to 139219.26)	1286917992 (1146820963 to 1436705137)	115140.51 (104963.9 to 126783.5)	-0.09 (-0.13 to -0.03)
	Deaths	971623 (769772 to 1199430)	226.43 (166.82 to 311.5)	547373 (414232 to 713744)	75.25 (50.89 to 110.13)	-0.68 (-0.76 to -0.6)
	DALYs	69215477 (53961045 to 83956627)	9231.04 (7207.31 to 11730.12)	36111927 (27436121 to 45426990)	2999.43 (2315.53 to 3859.13)	-0.7 (-0.77 to -0.63)
GBD Regions						
Andean Latin America	Prevalence	637098 (587004 to 692561)	1438.45 (1338.61 to 1549.33)	225849 (198333 to 258588)	347.21 (306.64 to 396.98)	-0.76 (-0.78 to -0.73)
	Incidence	37003808 (33077888 to 41140431)	83887.59 (75901.95 to 92599.28)	15483051 (13322848 to 18133567)	23820.04 (20605.46 to 27817.86)	-0.72 (-0.74 to -0.69)
	Deaths	10189 (8652 to 12022)	24.64 (20.18 to 29.42)	1717 (1202 to 2643)	2.92 (2.03 to 4.47)	-0.88 (-0.92 to -0.84)
	DALYs	859437 (739200 to 998145)	1722.02 (1478.56 to 1997.06)	106991 (80792 to 142675)	172.7 (129.89 to 229.98)	-0.9 (-0.92 to -0.87)
Australasia	Prevalence	54259 (46462 to 62444)	275.84 (234.87 to 318.67)	28194 (24877 to 31910)	86 (74.09 to 100.08)	-0.69 (-0.71 to -0.66)
	Incidence	3633258 (3089858 to 4214597)	18410.12 (15461.84 to 21460.9)	1852252 (1607504 to 2114402)	5649.98 (4834.1 to 6567.15)	-0.69 (-0.72 to -0.67)
	Deaths	144 (130 to 156)	0.68 (0.62 to 0.75)	300 (247 to 334)	0.5 (0.42 to 0.55)	-0.29 (-0.36 to -0.21)
	DALYs	9454 (7556 to 12101)	48.26 (38.35 to 61.59)	7528 (6479 to 8784)	19.75 (16.68 to 23.93)	-0.6 (-0.63 to -0.56)
Caribbean	Prevalence	205617 (182351 to 231442)	545.92 (487.37 to 610.99)	197728 (172514 to 226101)	431.58 (375.13 to 496.73)	-0.21 (-0.26 to -0.17)
	Incidence	13526296 (11668609 to 15430483)	35922.73 (31233.84 to 40827.91)	13633676 (11685295 to 16054870)	29747.59 (25321.07 to 35131.8)	-0.17 (-0.23 to -0.12)
	Deaths	14784 (12439 to 17311)	37.84 (32.09 to 44.4)	5867 (4156 to 7944)	14.02 (9.89 to 18.88)	-0.63 (-0.74 to -0.52)
	DALYs	1218184 (1013446 to 1427693)	2924.35 (2452.1 to 3433.32)	432887 (308278 to 575802)	1090.49 (769.64 to 1461.56)	-0.63 (-0.74 to -0.51)
Central Asia	Prevalence	371269 (336412 to 410692)	426.91 (386.5 to 473.53)	136164 (115852 to 158872)	141.7 (120.12 to 165.64)	-0.67 (-0.7 to -0.64)
	Incidence	22127122 (19231924 to 25016511)	25482 (22210.82 to 28897.23)	8500235 (7102605 to 9994522)	8829.8 (7339.39 to 10394.51)	-0.65 (-0.68 to -0.62)
	Deaths	14303 (12955 to 15923)	15.52 (14.08 to 17.26)	1929 (1428 to 2578)	1.96 (1.46 to 2.62)	-0.87 (-0.91 to -0.83)
	DALYs	1295085 (1171023 to 1440412)	1384.22 (1252.12 to 1538.59)	179360 (135239 to 237289)	181.19 (136.74 to 239.6)	-0.87 (-0.9 to -0.83)
Central Europe	Prevalence	59402 (48041 to 71969)	61.08 (49.9 to 73.7)	31447 (27856 to 35322)	31.03 (25.9 to 36.63)	-0.49 (-0.52 to -0.46)
	Incidence	3524216 (2801998 to 4306524)	3606.12 (2852.61 to 4421.83)	1918889 (1671273 to 2151872)	1940.64 (1588.77 to 2317.59)	-0.46 (-0.49 to -0.43)
	Deaths	1101 (1003 to 1210)	1.11 (1 to 1.23)	2587 (2291 to 2857)	1.38 (1.23 to 1.52)	0.18 (0.04 to 0.32)
	DALYs	85107 (77245 to 94006)	92.63 (83.37 to 102.36)	58242 (52321 to 64426)	53.61 (47.2 to 60.41)	-0.44 (-0.53 to -0.35)
Central Latin America	Prevalence	1685320 (1475073 to 1930555)	922.97 (824.47 to 1033.36)	821783 (731603 to 924682)	336.52 (298.71 to 379.58)	-0.64 (-0.65 to -0.62)
	Incidence	106731052 (91671723 to 122913384)	58634.62 (51678.52 to 66144.45)	54272972 (47604897 to 62316837)	22249.12 (19462.14 to 25539.5)	-0.62 (-0.64 to -0.6)
	Deaths	57020 (53692 to 60966)	35.7 (33.87 to 37.7)	12001 (10339 to 14016)	5.29 (4.52 to 6.22)	-0.86 (-0.88 to -0.83)
	DALYs	4383927 (4102666 to 4728530)	2095.79 (1973.25 to 2244.48)	585977 (491974 to 708152)	265.96 (220.2 to 324.16)	-0.88 (-0.9 to -0.85)
Central Sub-Saharan Africa	Prevalence	1076393 (988522 to 1171299)	1480.01 (1370.45 to 1598.41)	1944566 (1788238 to 2126501)	1387.7 (1298 to 1486.26)	-0.07 (-0.12 to -0.01)
	Incidence	64737365 (57444092 to 72181916)	90480.93 (81487.72 to 100113.35)	133600151 (119153900 to 151351998)	94579.16 (86007.3 to 105129.88)	0.04 (-0.03 to 0.13)
	Deaths	85579 (62473 to 104671)	148.47 (100.81 to 202.43)	35116 (23282 to 50781)	43.08 (25.65 to 67.35)	-0.73 (-0.82 to -0.64)
	DALYs	6703204 (4870684 to 8229530)	7184.05 (5299.52 to 8890.93)	2394531 (1678182 to 3358268)	1728.05 (1182.63 to 2452.9)	-0.78 (-0.84 to -0.71)
East Asia	Prevalence	4892517 (4147855 to 5808512)	413.68 (349.66 to 491.17)	1523826 (1294326 to 1772754)	116.74 (97.8 to 137.63)	-0.72 (-0.74 to -0.69)
	Incidence	297864969 (246384420 to 352693370)	25263.17 (20941.29 to 29838.11)	101274027 (85606904 to 118448050)	7713.32 (6387.53 to 9177.52)	-0.69 (-0.72 to -0.67)
	Deaths	95230 (70931 to 117616)	9.01 (6.5 to 11.39)	5893 (4191 to 9552)	0.44 (0.33 to 0.63)	-0.95 (-0.97 to -0.94)
	DALYs	7989901 (6186017 to 9826256)	681.69 (526.2 to 838.12)	406593 (329152 to 510531)	36.88 (29.99 to 45.96)	-0.95 (-0.96 to -0.93)
Eastern Europe	Prevalence	740072 (608559 to 880252)	398.84 (327.17 to 474.49)	350071 (288904 to 422655)	214.2 (171.28 to 262.11)	-0.46 (-0.51 to -0.41)
	Incidence	45627064 (36790586 to 55097865)	24587.31 (19703.28 to 29834.95)	21782627 (17510047 to 26437234)	13426.49 (10585.13 to 16628.67)	-0.45 (-0.5 to -0.4)
	Deaths	2100 (2024 to 2182)	1.16 (1.12 to 1.21)	585 (545 to 623)	0.26 (0.24 to 0.27)	-0.79 (-0.8 to -0.77)
	DALYs	235178 (206399 to 272360)	136.26 (120.42 to 155.99)	61076 (47513 to 77854)	38.44 (29.69 to 49.29)	-0.72 (-0.76 to -0.68)
Eastern Sub-Saharan Africa	Prevalence	5119633 (4664167 to 5639418)	2183.19 (2022.55 to 2381.52)	7720002 (7085740 to 8421769)	1787.88 (1666.27 to 1916.12)	-0.19 (-0.23 to -0.15)
	Incidence	308972113 (274106477 to 347935023)	133773.19 (120477.21 to 149066.93)	504500793 (449626298 to 568451319)	116696.88 (106204.64 to 129070.34)	-0.13 (-0.18 to -0.08)
	Deaths	317581 (231951 to 413916)	180.6 (113.91 to 263.03)	164018 (117642 to 218541)	62.72 (37.23 to 84.34)	-0.67 (-0.78 to -0.49)
	DALYs	23878766 (16853365 to 31040384)	8098.06 (5797.76 to 10825.01)	11045655 (8363694 to 14313440)	2523.08 (1853.83 to 3295.26)	-0.71 (-0.79 to -0.6)
High-income Asia Pacific	Prevalence	696565 (568274 to 836214)	458.84 (373.48 to 556.14)	829379 (728360 to 942444)	585.88 (486.35 to 692.85)	0.28 (0.18 to 0.37)
	Incidence	41653270 (33869000 to 49621246)	27758.11 (22376.07 to 33584.45)	53657289 (46068548 to 61883794)	37568.18 (30699.1 to 44976.2)	0.35 (0.28 to 0.44)
	Deaths	1644 (1318 to 1943)	1.08 (0.87 to 1.26)	4841 (3697 to 6024)	0.8 (0.64 to 1)	-0.27 (-0.38 to -0.11)
	DALYs	122703 (96255 to 156214)	83.7 (64.81 to 107.43)	157361 (127042 to 198469)	85.92 (63.03 to 115.97)	0.02 (-0.09 to 0.12)
High-income North America	Prevalence	410367 (329027 to 515651)	160.16 (126.76 to 203.36)	71763 (64432 to 79478)	18.67 (16.47 to 21.07)	-0.88 (-0.9 to -0.87)
	Incidence	26187750 (20691270 to 32549003)	10166.2 (7966.47 to 12788)	4348391 (3822662 to 4943141)	1125.19 (978.91 to 1297.34)	-0.89 (-0.9 to -0.87)
	Deaths	959 (874 to 1014)	0.28 (0.26 to 0.29)	10322 (8838 to 11150)	1.49 (1.3 to 1.6)	3.85 (3.51 to 4.08)
	DALYs	81130 (64472 to 100818)	30.54 (23.92 to 38.41)	180935 (164342 to 191982)	32.31 (29.91 to 34.2)	0.02 (-0.19 to 0.27)
North Africa and Middle East	Prevalence	3221544 (2798552 to 3668830)	686.35 (598.76 to 779.37)	2874392 (2409657 to 3421853)	456.42 (385.05 to 541.43)	-0.34 (-0.4 to -0.28)
	Incidence	193045883 (161958092 to 225157945)	41622.41 (35195.23 to 48177.47)	192887301 (158892555 to 234109970)	30655.52 (25356.16 to 36889.09)	-0.26 (-0.33 to -0.19)
	Deaths	101940 (78959 to 127704)	21.87 (16.67 to 27.16)	18339 (13735 to 24094)	3.53 (2.62 to 4.75)	-0.85 (-0.88 to -0.81)
	DALYs	9063178 (7068838 to 11340027)	1745.34 (1343.31 to 2182.98)	1656313 (1271459 to 2170155)	280.4 (216.48 to 367.29)	-0.85 (-0.88 to -0.81)
Oceania	Prevalence	88853 (81463 to 97451)	1144.13 (1055.39 to 1240.41)	148580 (136730 to 162855)	1014.26 (942.88 to 1094.23)	-0.15 (-0.19 to -0.1)
	Incidence	5484843 (4914907 to 6137868)	72521.57 (65043.68 to 80161.63)	10063172 (9077770 to 11227900)	68839.7 (62561.85 to 75931.16)	-0.06 (-0.12 to 0)
	Deaths	3757 (2615 to 5047)	79.04 (48.81 to 109.52)	4027 (2769 to 5418)	43.48 (27.78 to 62.25)	-0.5 (-0.59 to -0.4)
	DALYs	267031 (190767 to 363055)	2926.72 (2062.22 to 3889.14)	269613 (184474 to 376343)	1780.07 (1253.7 to 2384.7)	-0.49 (-0.6 to -0.38)
South Asia	Prevalence	24678581 (21701853 to 27927901)	1971.36 (1770.28 to 2195.22)	34162384 (31044486 to 37491065)	1878.93 (1716.49 to 2049.84)	-0.09 (-0.14 to -0.04)
	Incidence	1550617053 (1320986591 to 1762161539)	128123.11 (112430.41 to 144337.51)	2215979139 (1971336860 to 2476296089)	121872.67 (108949 to 135347.48)	-0.06 (-0.12 to 0.01)
	Deaths	1664505 (1330406 to 2176400)	230.96 (169.95 to 330.02)	650729 (430032 to 1010207)	52.25 (34.34 to 81.39)	-0.79 (-0.84 to -0.72)
	DALYs	97275211 (80465643 to 117199267)	7630.49 (6111.88 to 9999.63)	26267550 (20172537 to 35803484)	1688.36 (1282.69 to 2324.84)	-0.8 (-0.84 to -0.76)
Southeast Asia	Prevalence	7141453 (6258765 to 8098329)	1369.55 (1218.49 to 1531.94)	5449156 (4951789 to 6076444)	829.83 (747.92 to 929)	-0.41 (-0.44 to -0.38)
	Incidence	423236909 (362813623 to 483950260)	82572.88 (72303.9 to 93680.92)	358637500 (313989689 to 402974774)	54498.27 (47651.09 to 61254.88)	-0.34 (-0.37 to -0.31)
	Deaths	346228 (240210 to 488465)	98.38 (57.22 to 152.66)	88987 (53789 to 116713)	16.32 (9.52 to 21.72)	-0.84 (-0.9 to -0.76)
	DALYs	22490665 (16325875 to 28930584)	4208.73 (2932.65 to 5788.35)	4232029 (3283180 to 5305626)	702.82 (539.9 to 881.87)	-0.85 (-0.88 to -0.8)
Southern Latin America	Prevalence	320724 (268393 to 380934)	634.7 (534.37 to 749.55)	118878 (99858 to 138670)	191.76 (158.87 to 226.28)	-0.7 (-0.72 to -0.68)
	Incidence	20533698 (17182877 to 24543776)	40675.52 (34101.07 to 48467.86)	7912146 (6527708 to 9292111)	12762.35 (10398.97 to 15130.27)	-0.69 (-0.71 to -0.67)
	Deaths	1638 (1547 to 1719)	3.49 (3.29 to 3.67)	1267 (1122 to 1403)	1.53 (1.36 to 1.69)	-0.57 (-0.61 to -0.53)
	DALYs	130865 (116790 to 146123)	258.17 (230.11 to 288.49)	41123 (36017 to 47869)	65.46 (56.41 to 77.33)	-0.75 (-0.78 to -0.72)
Southern Sub-Saharan Africa	Prevalence	1573635 (1401466 to 1761372)	2707.46 (2432.39 to 2994.73)	1372608 (1255656 to 1500758)	1688.18 (1552.89 to 1834.68)	-0.38 (-0.41 to -0.34)
	Incidence	96530924 (84596034 to 109166428)	166237.52 (148057.31 to 188063.74)	89893938 (79721355 to 102583407)	110703.56 (98915.34 to 125610.4)	-0.33 (-0.37 to -0.28)
	Deaths	38729 (32139 to 47502)	82.24 (60.05 to 116.63)	24462 (17182 to 33542)	40.58 (26.73 to 58.19)	-0.52 (-0.6 to -0.4)
	DALYs	2875263 (2497611 to 3307024)	4453.58 (3800.92 to 5350.47)	1451876 (1163483 to 1831288)	1941.05 (1555.22 to 2486.17)	-0.57 (-0.64 to -0.49)
Tropical Latin America	Prevalence	1263454 (1081971 to 1474675)	777.8 (675.36 to 894.8)	604160 (525832 to 693384)	284.37 (244.36 to 330.11)	-0.63 (-0.66 to -0.61)
	Incidence	78226903 (64938932 to 92548183)	48190.25 (40896.06 to 56253.16)	41643472 (35593321 to 48562339)	19491.36 (16521.16 to 22919.92)	-0.6 (-0.62 to -0.57)
	Deaths	38029 (33520 to 42625)	27.72 (25.04 to 30.59)	6243 (5475 to 6721)	2.71 (2.37 to 2.92)	-0.9 (-0.91 to -0.89)
	DALYs	3154322 (2730620 to 3582444)	2006.05 (1745.51 to 2268.63)	244093 (216599 to 276067)	116.69 (102.98 to 133.14)	-0.94 (-0.95 to -0.93)
Western Europe	Prevalence	1408001 (1183959 to 1689714)	438.6 (355.04 to 539.24)	1683577 (1496791 to 1899296)	416.16 (345.33 to 490.54)	-0.05 (-0.11 to 0.01)
	Incidence	92167543 (76349297 to 110346130)	28505.1 (22757.4 to 34939.87)	102588935 (89592335 to 116188594)	25442.59 (20832.64 to 30200.77)	-0.11 (-0.15 to -0.07)
	Deaths	2980 (2680 to 3170)	0.52 (0.47 to 0.56)	13318 (10985 to 14776)	1.12 (0.95 to 1.23)	0.96 (0.81 to 1.09)
	DALYs	216573 (163747 to 282965)	63.23 (45.71 to 84.64)	358703 (300485 to 429953)	68.24 (52.83 to 88.15)	0.05 (-0.01 to 0.13)
Western Sub-Saharan Africa	Prevalence	5410647 (4885111 to 5933268)	2363.89 (2169.09 to 2568.62)	7532094 (6865406 to 8320158)	1482.84 (1372.3 to 1604.33)	-0.38 (-0.41 to -0.35)
	Incidence	327227137 (288708266 to 366253411)	144649.34 (130049.86 to 162001.6)	513977912 (454637135 to 578613767)	101090.39 (91655.59 to 111930.73)	-0.3 (-0.34 to -0.26)
	Deaths	416168 (304048 to 507528)	188.92 (127.39 to 258.31)	283674 (203307 to 375380)	65.53 (45.98 to 88.84)	-0.68 (-0.75 to -0.59)
	DALYs	32103726 (23334866 to 38990208)	9643.34 (6926.45 to 11864.65)	21790570 (15281983 to 29569603)	3383.88 (2464.9 to 4393.17)	-0.68 (-0.75 to -0.6)

DALYs, Disability-adjusted life years; GBD, Global Burden of Disease.

#### Diarrheal diseases

In 2021, the global prevalence was 67,276,401 with an ASPR of 872.02; from 1990 to 2021, the ASPR decreased by 0.16. The number of incident cases was 4,438,577,275 with an ASIR of 57,586.1; the ASIR decreased by 0.12 from 1990 to 2021. The number of deaths was 1,165,398 with an ASMR of 15.42; the ASMR decreased by 0.75 during the same period. The global DALYs totaled 58,983,817 with an ASR of 834.18, and the ASR of DALYs decreased by 0.75 from 1990 to 2021 (**Supplementary Table S1**).

#### Typhoid fever

In 2021, the global prevalence was 452,343 with an ASPR of 6.23. From 1990 to 2021, the ASPR decreased by 0.7. The number of incident cases was 715,455 with an ASIR of 98.56, and the ASIR decreased by 0.7 over this period. The number of deaths was 93,333 with an ASMR of 1.31, which decreased by 0.59 from 1990 to 2021. The global DALYs were 7,087,733 with an ASR of DALYs of 101.09, and the ASR of DALYs decreased by 0.59 during this period (**Supplementary Table S2**).

#### Paratyphoid fever

In 2021, the global prevalence was 94,953 with an ASPR of 1.28. Between 1990 and 2021, the ASPR decreased by 0.79. The number of incident cases was 2,166,063 with an ASIR of 29.21; the ASPR decreased by 0.79 from 1990 to 2021. The number of deaths was 14,127 with an ASMR of 0.19, and the ASMR decreased by 0.72 over the same period. The global DALYs totaled 1,011,842 with an ASR of DALYs of 14.16, which decreased by 0.73 from 1990 to 2021 (**Supplementary Table S3**).

#### iNTS

In 2021, the global prevalence was 11,847, with an ASPR of 0.17. From 1990 to 2021, the ASPR increased by 0.21. The number of incident cases was 509,976 with an ASIR of 7.21; the ASIR increased by 0.2 over the same period. The number of deaths was 62,018 with an ASMR of 0.88, and the ASMR increased by 0.02 from 1990 to 2021. The global DALYs were 4,740,235, with an ASR of DALYs of 69.14, and the ASR of DALYs increased by 0.12 during this period (**Supplementary Table S4**).

### Regional level

#### Enteric infections

**Figure 1:**
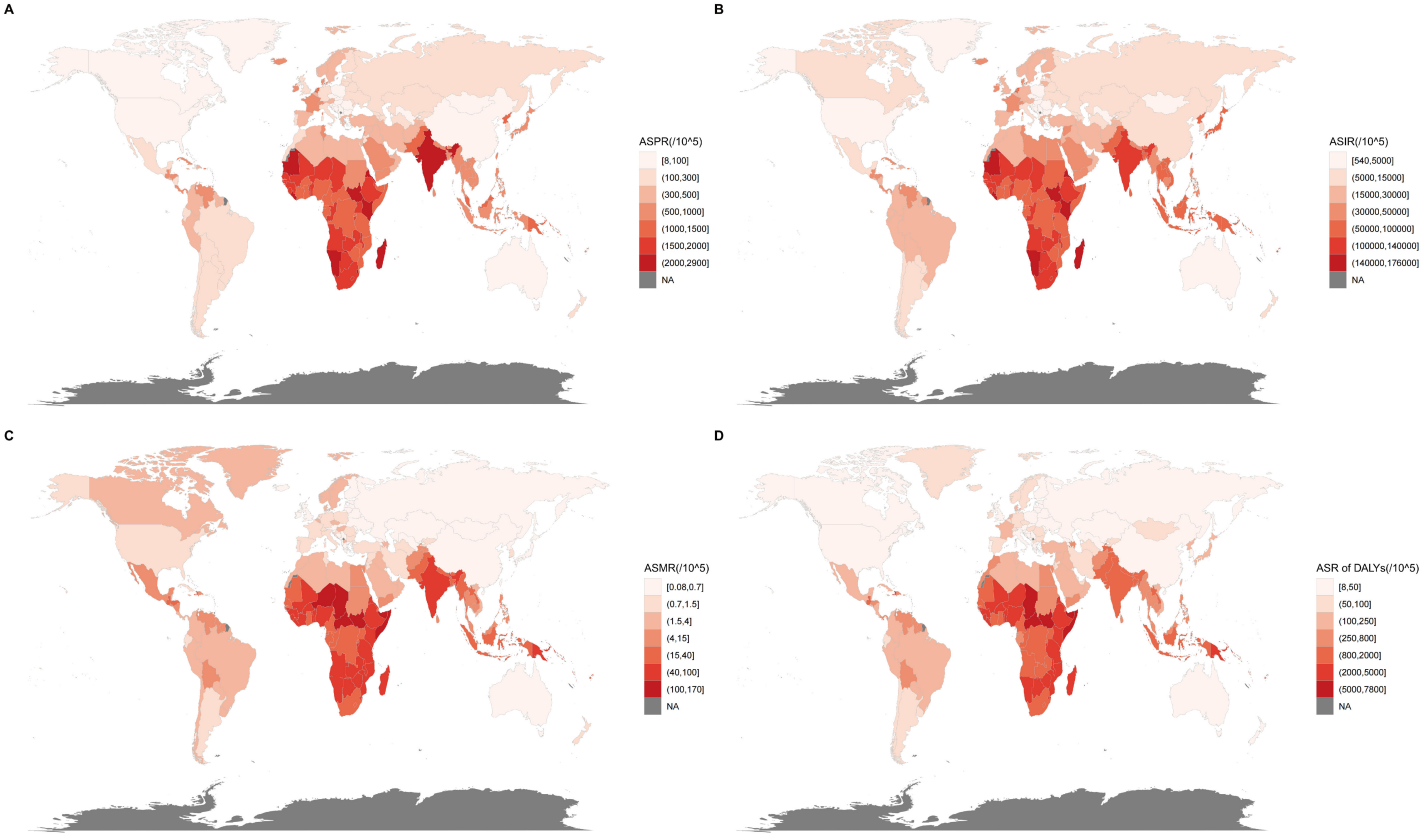
Global distribution of enteric infections disease burden in 2021. **(A)** ASPR of enteric infections; **(B)** ASIR of enteric infections; **(C)** ASMR of enteric infections; **(D)** ASR of DALYs of enteric infections. ASPR, Age-standardized prevalence rates; ASIR, Age-standardized incidence rates; ASMR, Age-standardized mortality rates; ASR, Age-standardized rate; DALYs, Disability-adjusted life years.

In 2021, the ASPR was highest in Low SDI regions, reaching 1774.15. Geographically, the South Asia region exhibited the highest ASPR at 1878.93 (**Table 1**; **Figure 1A**). From 1990 to 2021, the slowest decline was observed in Low SDI regions, with a decrease of 0.14. Geographically, the High-income Asia Pacific region showed the most significant upward trend, at 0.28 (**Table 1**). The ASIR was highest in Low SDI regions, at 115140.51, with South Asia again exhibiting the highest ASIR at 121872.67 (**Table 1**; **Figure 1B**). Between 1990 and 2021, the slowest decline was observed in Low SDI regions, with a downward trend of 0.09. Geographically, the High-income Asia Pacific region showed the most significant upward trend, at 0.35 (**Table 1**). The ASMR was highest in Low SDI regions, at 75.25. Geographically, the Western Sub-Saharan Africa region had the highest ASMR at 65.53 (**Table 1**; **Figure 1C**). Between 1990 and 2021, the fastest increase was observed in High SDI regions, with an upward trend of 0.25. Geographically, the High-income North America region showed the most significant upward trend, at 3.85 (**Table 1**). The ASR of DALYs was highest in Low SDI regions, at 2999.43. Geographically, the Western Sub-Saharan Africa region had the highest ASR of DALYs at 3383.88 (**Table 1**; **Figure 1D**). Between 1990 and 2021, the slowest decline was observed in High SDI regions, with a downward trend of 0.3. Geographically, Western Europe showed the most significant upward trend, at 0.05 (**Table 1**).

#### Diarrheal diseases

In 2021, the ASPR was highest in Low SDI regions, at 1763.73. Geographically, the South Asia region had the highest ASPR at 1857.37. Between 1990 and 2021, the slowest decline was observed in Low SDI regions, with a downward trend of 0.13. Geographically, the High-income Asia Pacific region showed the most significant upward trend, at 0.28 (**Supplementary Table S1**). The ASIR was highest in Low SDI regions, at 114948.62. Geographically, the South Asia region had the highest ASIR at 121489.61. Between 1990 and 2021, the slowest decline was observed in Low SDI regions, with a downward trend of 0.08. Geographically, the High-income Asia Pacific region showed the most significant upward trend, at 0.35 (**Supplementary Table S1**). The ASMR was highest in Low SDI regions, at 69.75. Geographically, the Eastern Sub-Saharan Africa region had the highest ASMR at 59.99. Between 1990 and 2021, the fastest increase was observed in High SDI regions, with an upward trend of 0.35. Geographically, the High-income North America region showed the most significant upward trend, at 4.36 (**Supplementary Table S1**). The ASR of DALYs was highest in Low SDI regions, at 2605.84. Geographically, the Western Sub-Saharan Africa region had the highest ASR of DALYs at 2769.81. Between 1990 and 2021, the slowest decline was observed in High SDI regions, with a downward trend of 0.27. Geographically, Western Europe showed the most significant upward trend, at 0.08 (**Supplementary Table S1**).

#### Typhoid fever

The ASPR was highest in Low-middle SDI regions, at 12.09. Geographically, the South Asia region had the highest ASPR at 17.01. Between 1990 and 2021, the slowest decline was observed in High-middle SDI regions, with a downward trend of 0.54. Geographically, Australasia showed the least decline, at 0.01 (**Supplementary Table S2**). The ASIR was highest in Low-middle SDI regions, at 191.27. Geographically, the South Asia region had the highest ASIR at 269.08. Between 1990 and 2021, the slowest decline was observed in High-middle SDI regions, with a downward trend of 0.54. Geographically, Australasia showed the least decline, at 0.01 (**Supplementary Table S2**). The ASMR was highest in Low-middle SDI regions, at 2.52. Geographically, the Oceania region had the highest ASMR at 3.48. Between 1990 and 2021, the slowest decline was observed in High-middle SDI regions, with a downward trend of 0.57. Geographically, Australasia showed the most significant upward trend, at 0.71 (**Supplementary Table S2**). The ASR of DALYs was highest in Low-middle SDI regions, at 188.51. Geographically, the South Asia region had the highest ASR of DALYs at 260.27. Between 1990 and 2021, the slowest decline was observed in High-middle SDI regions, with a downward trend of 0.58. Geographically, Southern Sub-Saharan Africa showed the most significant upward trend, at 0.03 (**Supplementary Table S2**).

#### Paratyphoid fever

In 2021, the ASPR was highest in Low-middle SDI regions, at 2.93. Geographically, the South Asia region had the highest ASPR at 4.85. Between 1990 and 2021, the slowest decline was observed in High SDI regions, with a downward trend of 0.33. Geographically, Australasia showed the most significant upward trend, at 0.62 (**Supplementary Table S3**). The ASIR was highest in Low-middle SDI regions, at 66.83. Geographically, the South Asia region had the highest ASIR at 110.56. Between 1990 and 2021, the slowest decline was observed in High SDI regions, with a downward trend of 0.34. Geographically, Australasia showed the most significant upward trend, at 0.61 (**Supplementary Table S3**). The ASMR was highest in Low-middle SDI regions, at 0.44. Geographically, the South Asia region had the highest ASMR at 0.73. Between 1990 and 2021, the slowest decline was observed in High-middle SDI regions, with a downward trend of 0.67. Geographically, High-income North America showed the most significant upward trend, at 0.73 (**Supplementary Table S3**). The ASR of DALYs was highest in Low-middle SDI regions, at 30.99. Geographically, the South Asia region had the highest ASR of DALYs at 51.64. Between 1990 and 2021, the slowest decline was observed in High-middle SDI regions, with a downward trend of 0.69. Geographically, High-income North America showed the most significant upward trend, at 0.54 (**Supplementary Table S3**).

#### iNTS

In 2021, the ASPR was highest in Low SDI regions, at 0.48. Geographically, the Western Sub-Saharan Africa region had the highest ASPR at 1.1. Between 1990 and 2021, the fastest increase was observed in High SDI regions, with an upward trend of 0.18. Geographically, Western Europe showed the most significant upward trend, at 0.68 (**Supplementary Table S4**). The ASIR was highest in Low SDI regions, at 20.91. Geographically, the Western Sub-Saharan Africa region had the highest ASIR at 47.54. Between 1990 and 2021, the fastest increase was observed in High SDI regions, with an upward trend of 0.17. Geographically, Western Europe showed the most significant upward trend, at 0.66 (**Supplementary Table S4**). The ASMR was highest in Low SDI regions, at 3.3. Geographically, the Western Sub-Saharan Africa region had the highest ASMR at 6.88. Between 1990 and 2021, the slowest decline was observed in Low SDI regions, with a downward trend of 0.31. Geographically, the Caribbean showed the most significant upward trend, at 0.01 (**Supplementary Table S4**). The ASR of DALYs was highest in Low SDI regions, at 234.06. Geographically, the Western Sub-Saharan Africa region had the highest ASR of DALYs at 486.81. Between 1990 and 2021, the slowest decline was observed in Low SDI regions, with a downward trend of 0.24. Geographically, Oceania showed the most significant upward trend, at 0.06 (**Supplementary Table S4**).

### National and territorial level

#### Enteric infections

In 2021, India had the highest number of prevalence cases, totaling 29,470,511 (**Supplementary Table S5**). South Sudan had the highest ASPR at 2888.11 (**Supplementary Table S6**; **Figure 1A**). Between 1990 and 2021, North Korea experienced the fastest increase, with an upward trend of 2.68 (**Supplementary Table S7**). India had the highest number of incidence cases with 1,898,215,166 (**Supplementary Table S5**). South Sudan had the highest ASIR at 175498.06 (**Supplementary Table S6**; **Figure 1B**). Between 1990 and 2021, North Korea experienced the fastest increase, with an upward trend of 2.6 (**Supplementary Table S7**). Regarding deaths, India had the highest number with 552,102 (**Supplementary Table S5**). South Sudan had the highest ASMR at 168.95 (**Supplementary Table S6**; **Figure 1C**). Between 1990 and 2021, India experienced the fastest increase, with an upward trend of 15.37 (**Supplementary Table S7**). Furthermore, India also had the highest number of DALYs with 20,822,873 (**Supplementary Table S5**). Chad had the highest ASR of DALYs at 7791.23 (**Supplementary Table S6**; **Figure 1D**). Between 1990 and 2021, Sweden experienced the fastest increase, with an upward trend of 1.14 (**Supplementary Table S7**).

#### Diarrheal diseases

In 2021, India had the highest number of prevalence cases with 29,167,174 (**Supplementary Table S8**). South Sudan had the highest ASPR at 2881.95 (**Supplementary Table S9**). Between 1990 and 2021, North Korea experienced the fastest increase, with an upward trend of 2.69 (**Supplementary Table S10**). India had the highest number of incidence cases with 1,892,784,379 (**Supplementary Table S8**). South Sudan had the highest ASIR at 175395.84 (**Supplementary Table S9**). Between 1990 and 2021, North Korea experienced the fastest increase, with an upward trend of 2.6 (**Supplementary Table S10**). India also had the highest number of deaths with 496,725 (**Supplementary Table S8**). South Sudan had the highest ASMR at 166.68 (**Supplementary Table S9**). Between 1990 and 2021, Sweden experienced the fastest increase, with an upward trend of 16.15 (**Supplementary Table S10**). India had the highest number of DALYs with 16,762,076 (**Supplementary Table S8**). Chad had the highest ASR of DALYs at 7341.22 (**Supplementary Table S9**). Between 1990 and 2021, Sweden experienced the fastest increase, with an upward trend of 1.16 (**Supplementary Table S10**).

#### Typhoid fever

In 2021, a study conducted at the country and regional level found that India had the highest number of prevalence cases, incidence and deaths, with 234,912, 3,715,087 and 41,586 respectively (**Supplementary Table S11**). Burkina Faso had the highest ASPR at 20.76 (**Supplementary Table S12**). Between 1990 and 2021, Australia experienced the fastest increase, with an upward trend of 0.87 (**Supplementary Table S1**). Burkina Faso had the highest ASIR at 328.48 (**Supplementary Table S12**). Between 1990 and 2021, Australia experienced the fastest increase, with an upward trend of 0.86 (**Supplementary Table S1**). Bhutan had the highest ASMR at 5.61 (**Supplementary Table S12**). Denmark experienced the fastest increase, with an upward trend of 58.98 between 1990 and 2021 (**Supplementary Table S13**). India had the highest number of DALYs with 3,117,354 (**Supplementary Table S11**). Bhutan had the highest ASR of DALYs at 434.23 (**Supplementary Table S12**). Between 1990 and 2021, Denmark experienced the fastest increase, with an upward trend of 21.28 (**Supplementary Table S13**).

#### Paratyphoid fever

In 2021, India had the highest number of prevalence cases with 73,385 (**Supplementary Table S14**). India had the highest ASPR at 5.46 (**Supplementary Table S15**). Between 1990 and 2021, Kenya experienced the fastest increase, with an upward trend of 3.71 (**Supplementary Table S16**). India had the highest number of incidence cases with 1,673,611 (**Supplementary Table S14**). India also had the highest ASIR at 124.45 (**Supplementary Table S15**). Between 1990 and 2021, Kenya experienced the fastest increase, with an upward trend of 3.7 (**Supplementary Table S16**). India had the highest number of deaths with 9,893 (**Supplementary Table S14**). Pakistan had the highest ASMR at 1.05 (**Supplementary Table S15**). Between 1990 and 2021, Denmark experienced the fastest increase, with an upward trend of 275.01 (**Supplementary Table S16**). India also had the highest number of DALYs with 695,210 (**Supplementary Table S14**). Pakistan had the highest ASR of DALYs at 72.66 (**Supplementary Table S15**). Between 1990 and 2021, Denmark experienced the fastest increase, with an upward trend of 125.61 (**Supplementary Table S16**).

#### iNTS

In 2021, Nigeria had the highest number of prevalence cases with 3,844 (**Supplementary Table S17**). Mali had the highest ASPR at 1.74 (**Supplementary Table S18**). Between 1990 and 2021, the United Kingdom experienced the fastest increase, with an upward trend of 1.75 (**Supplementary Table S19**). Nigeria had the highest number of incidence cases with 164,230 (**Supplementary Table S17**). Mali had the highest ASIR at 76.24 (**Supplementary Table S18**). Between 1990 and 2021, the United Kingdom experienced the fastest increase, with an upward trend of 1.74 (**Supplementary Table S19**). Nigeria also had the highest number of deaths with 21,614 (**Supplementary Table S17**). Mali had the highest ASMR at 13.26 (**Supplementary Table S18**). Between 1990 and 2021, The Bahamas experienced the fastest increase, with an upward trend of 14.25 (**Supplementary Table S19**). Nigeria had the highest number of DALYs with 1,754,290 (**Supplementary Table S17**). Mali had the highest ASR of DALYs at 859.23 (**Supplementary Table S18**). Between 1990 and 2021, The Bahamas experienced the fastest increase, with an upward trend of 13.47 (**Supplementary Table S19**).

### Age, sex, and subtype analysis

**Figure 2:**
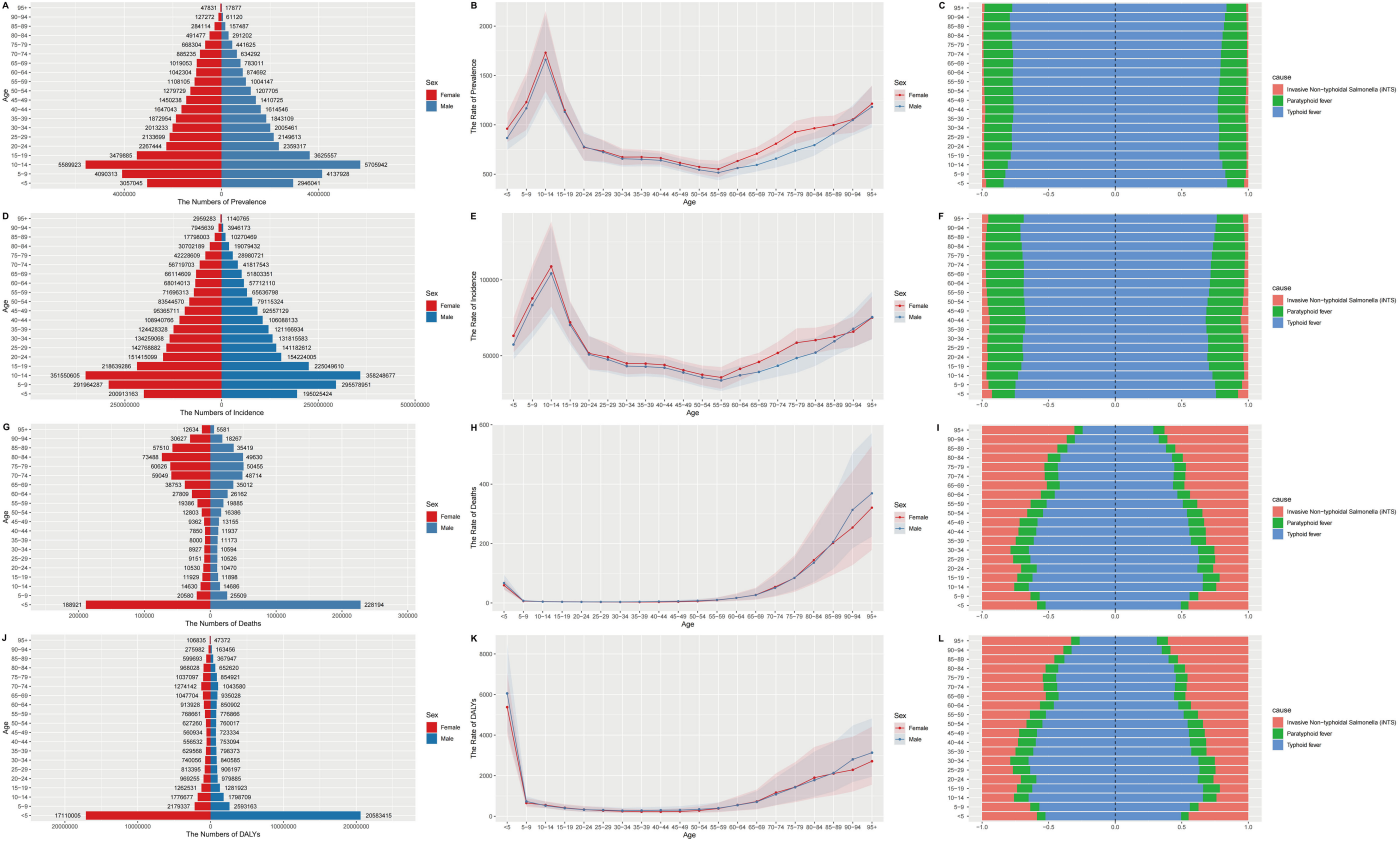
Sex, age-structured, and subtype analysis of enteric infections disease burden in 2021. **(A)** The number of prevalence; **(B)** The rate of prevalence; **(C)** The subtype of prevalence in different age groups; **(D)** The number of incidence; **(E)** The rate of incidence; **(F)** The subtype of incidence in different age groups; **(G)** The number of deaths; **(H)** The rate of deaths; **(I)** The subtype of deaths in different age groups; **(J)** The number of DALYs; **(K)** The rate of DALYs; **(L)** The subtype of DALYs in different age groups. DALYs, disability-adjusted life years.

In 2021, among enteric infections, the 10-14 year age group exhibited the highest prevalence and incidence rates, while mortality rate was highest among those aged 90 and above, and DALYs rate was highest in children under 5. Females under 90 had a higher burden of prevalence and incidence compared to males; however, for those aged over 90, both mortality and DALYs rates were lower in females than in males. Due to the large number and variety of pathogens causing diarrheal diseases, only three subtypes, typhoid fever, paratyphoid fever, and iNTS, were included for comparison (**Figure 2**). Typhoid fever had higher rates across all epidemiological indicators, with the largest proportion of cases seen in children under 5 for prevalence and incidence, iNTS in those over 85 for deaths and DALYs, and Paratyphoid fever had the lowest proportion under 10 years old. Regional distribution of subtypes varied, with the highest proportion of Typhoid fever in prevalence in North Africa and the Middle East having, the highest incidence rates in Eastern Sub-Saharan Africa, and the highest rates of deaths and DALYs in Southeast Asia (**Supplementary Figure S1**).

### Overall temporal trends

Globally, prevalence and incidence rose from 1990 to 2011, then declined until 2015. deaths and DALYs consistently declined, with a moderated decrease between 2003 and 2006(**Supplementary Figure S2**). In High SDI regions, prevalence and incidence rates shifted from a decline to an increase in 2019, while High-middle, Middle, and Low-middle SDI regions saw this change in 2000, 2016, and 2020, respectively. Low SDI regions had two periods of increase, from 2005 to 2009 and from 2018 to 2020. Except for High SDI peaking in 1991, all SDI regions showed a downward trend in deaths and DALYs. Among the 21 GBD regions, Southern Sub-Saharan Africa had the steepest decline in prevalence and Incidence rates, with females showing a significant increase in the rate of decline from 2004, and both genders showing an increase in 2019. Central Latin America saw a sharp decline in deaths and DALYs after an increase from 1995 to 2000 (**Supplementary Figure S3**).

### Association between ASR and SDI

**Figure 3:**
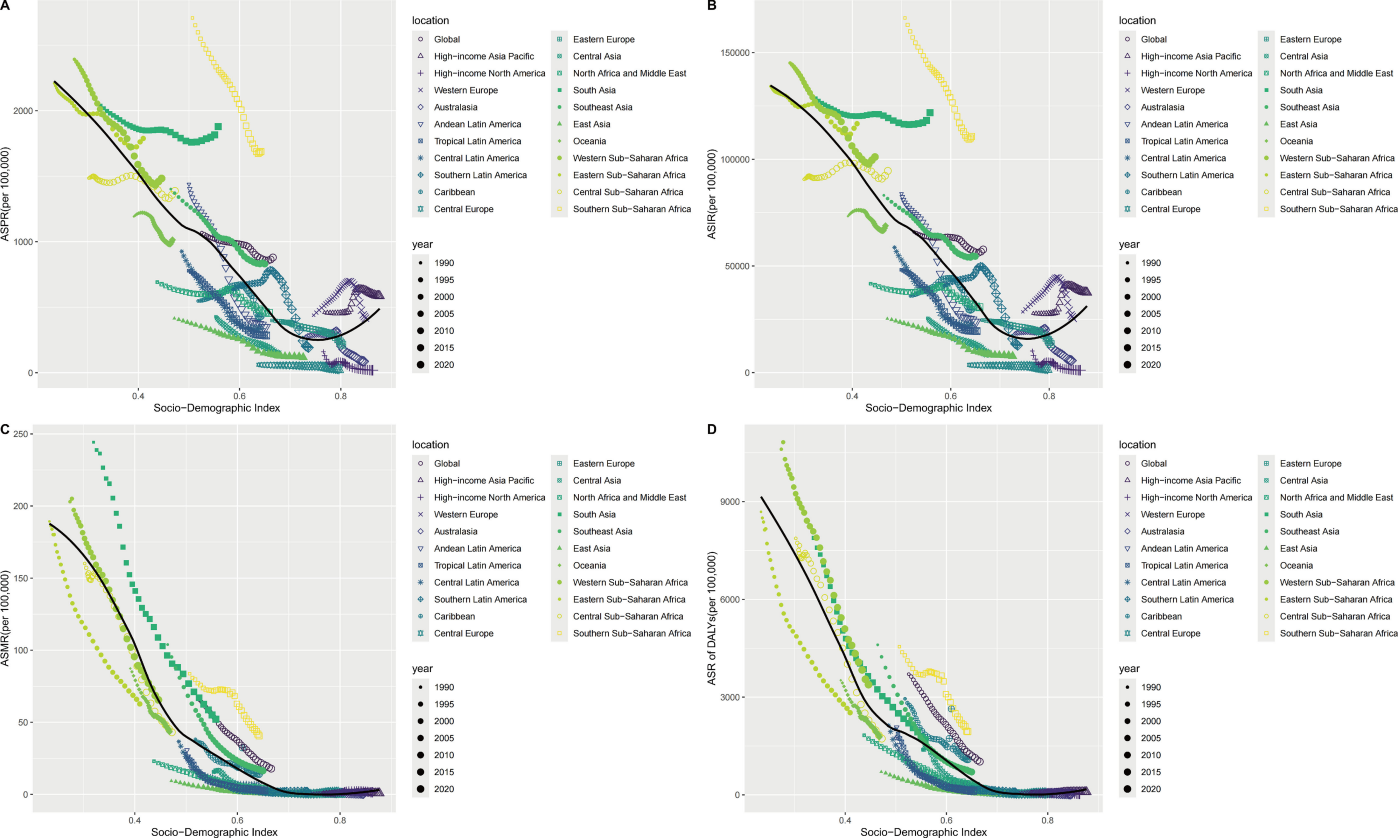
The association of the SDI and ASPR, ASIR ASMR, ASR of DALYs of enteric infections, globe and regions from 1990 to 2021. **(A)** The association of the SDI and ASPR; **(B)** The association of the SDI and ASIR; **(C)** The association of the SDI and ASMR; **(D)** The association of the SDI and ASR of DALYs. SDI, Socio-demographic index; ASPR, Age-standardized prevalence rates; ASIR, Age-standardized incidence rates; ASMR, Age-standardized mortality rates; ASR, Age-standardized rates; DALYs, Disability-adjusted life years.

This section primarily depicts the relationship between the four epidemiological indicators and the SDI. From 1990 to 2021, in the global context and across the 21 GBD regions, ASPR and ASIR declined with increasing SDI, but then rose again in High SDI regions. ASMR and ASR of DALYs also decreased with increasing SDI and remained at very low levels from High-middle SDI regions onwards (**Figure 3**). Analyzing 204 countries and regions in 2021, the overall trend is similar to that of the global and 21 GBD regions (**Supplementary Figure S4**).

### Risk factors analysis

**Figure 4:**
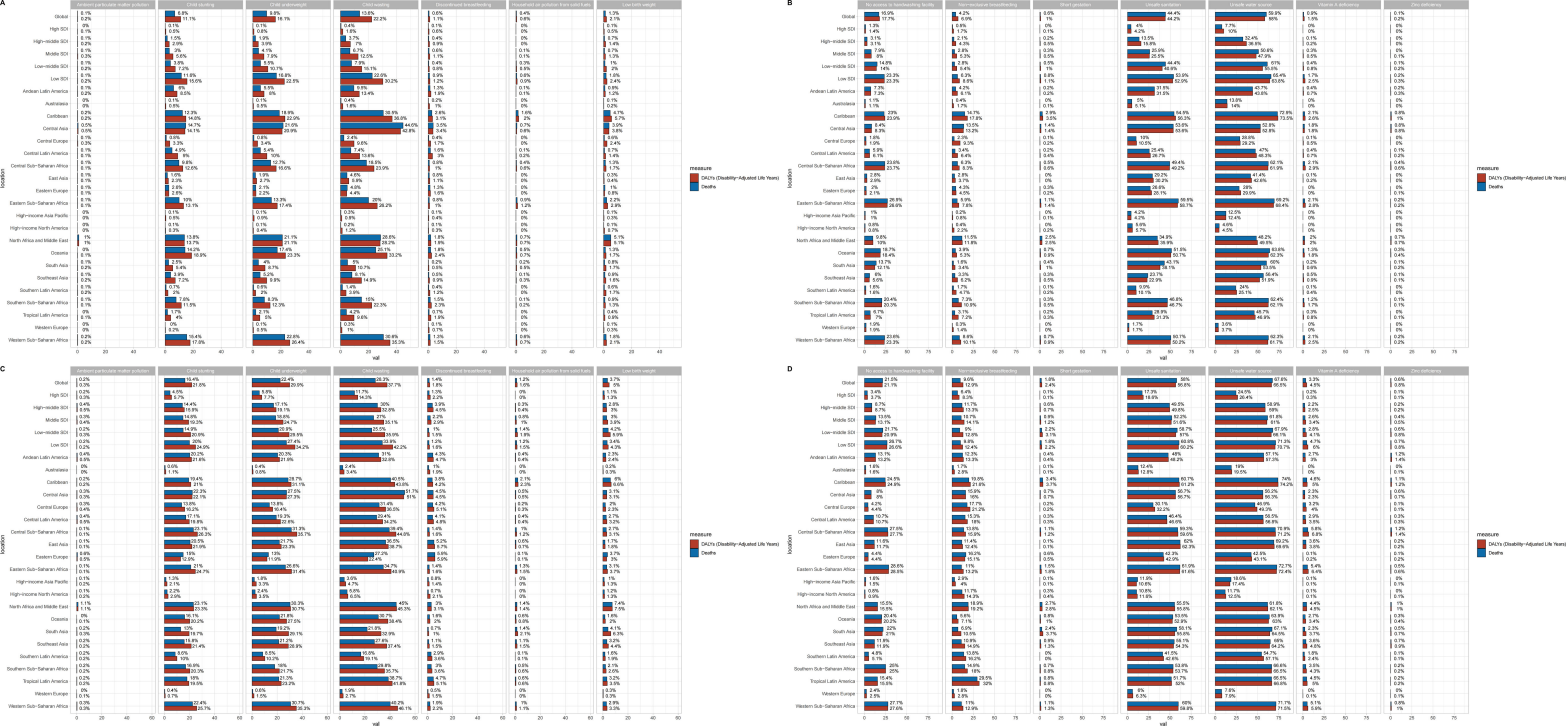
The enteric infections DALYs and deaths attributable to risk factors compared in 2021 and 1990, globally and by 21 GBD regions. **(A, B)** DALYs and deaths attributable to risk factors in 2021; (C&D) DALYs and deaths attributable to risk factors in 1990. DALYs, Disability-adjusted life years.

Globally, unsafe water source was the largest contributor to DALYs and deaths, with contribution rates of 58% and 59.9% respectively in 2021. In the 5 SDI regions, Low SDI regions had the largest proportion, with deaths and DALYs reaching 65.4% and 63.8%, while in High SDI regions, deaths and DALYs were only 7.7% and 10% respectively. Among the 21 GBD regions, the Caribbean had the largest proportion, with DALYs reaching up to 73.5%, while Western Europe had the lowest proportion, with Deaths at only 3.6% (**Figures 4A, B**). Other significant factors include unsafe sanitation, child wasting, child underweight, and child stunting, all of which were most prevalent in Low SDI regions (**Figure 4**). In 1990, the overall situation was similar to 2021, but each factor accounted for a significantly higher proportion (**Figures 4C, D**).

### Etiological analysis

**Figure 5:**
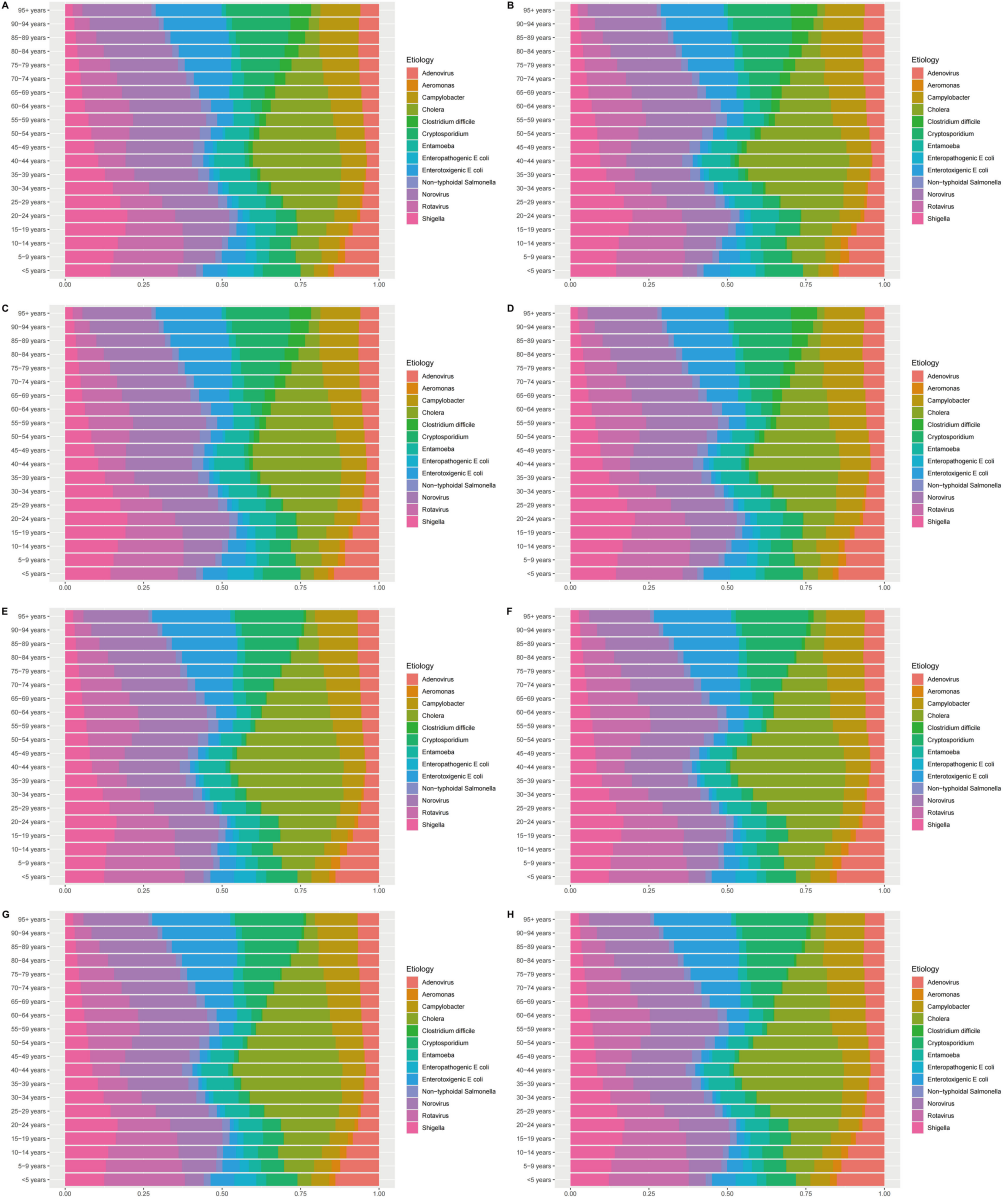
Etiological analysis of enteric infections for both sexes among all age groups in 1990 and 2021. **(A)** Etiological analysis of males in deaths (2021), **(B)** Etiological analysis of females in deaths (2021); **(C)** Etiological analysis of males in DALYs (2021); **(D)** Etiological analysis of females in DALYs (2021); **(E)** Etiological analysis of males in deaths (1990), **(F)** Etiological analysis of females in deaths (1990); **(G)** Etiological analysis of males in DALYs (1990); **(H)** Etiological analysis of females in DALYs (1990). DALYs, Disability-adjusted life years.

In 2021, Norovirus emerged as the primary cause of death from enteric infections across all age groups, peaking at 46.33 per 100,000 among individuals over 95. Rotavirus was identified as the main contributor to DALYs in children under 5, with a rate of 1741.65 per 100,000 in 2021, a significant decrease from the 1990 rate of 9398.04 per 100,000 (**Figure 5**; **Supplementary Tables S20**-**S32**). In 2021, Rotavirus was the leading cause of enteric infection fatalities globally, causing 92,607 deaths, with the highest number of total cases in Western Sub-Saharan Africa at 37,189 individuals. Among the five SDI regions, the Low SDI region experienced the highest number of Rotavirus-related deaths. In 1990, the Low-middle SDI region and South Asia had the highest death tolls, with 162,007 and 124,198 fatalities respectively. While Rotavirus has always been the most dangerous pathogen, Shigella was the third leading cause in 1990 with 17,500,460 cases, rising to the second-highest cause in 2021. (**Supplementary Figure S5**, **Supplementary Tables S33**-**S45** for the global, 5 SDI regions and 21 GBD regions, and **Supplementary Tables S46**-**S58** for 204 countries and territories).

Overall, there has been a significant decrease in the number of deaths and DALYs caused by various pathogens over the past 32 years. Globally, the number of deaths attributed to Rotavirus decreased from 392, 148 in 1990 to 92, 607 in 2021, and DALYs decreased from 32, 857,936 to 7, 279, 772, indicating a significant reduction in the number of cases.

### Decomposition analysis

**Figure 6:**
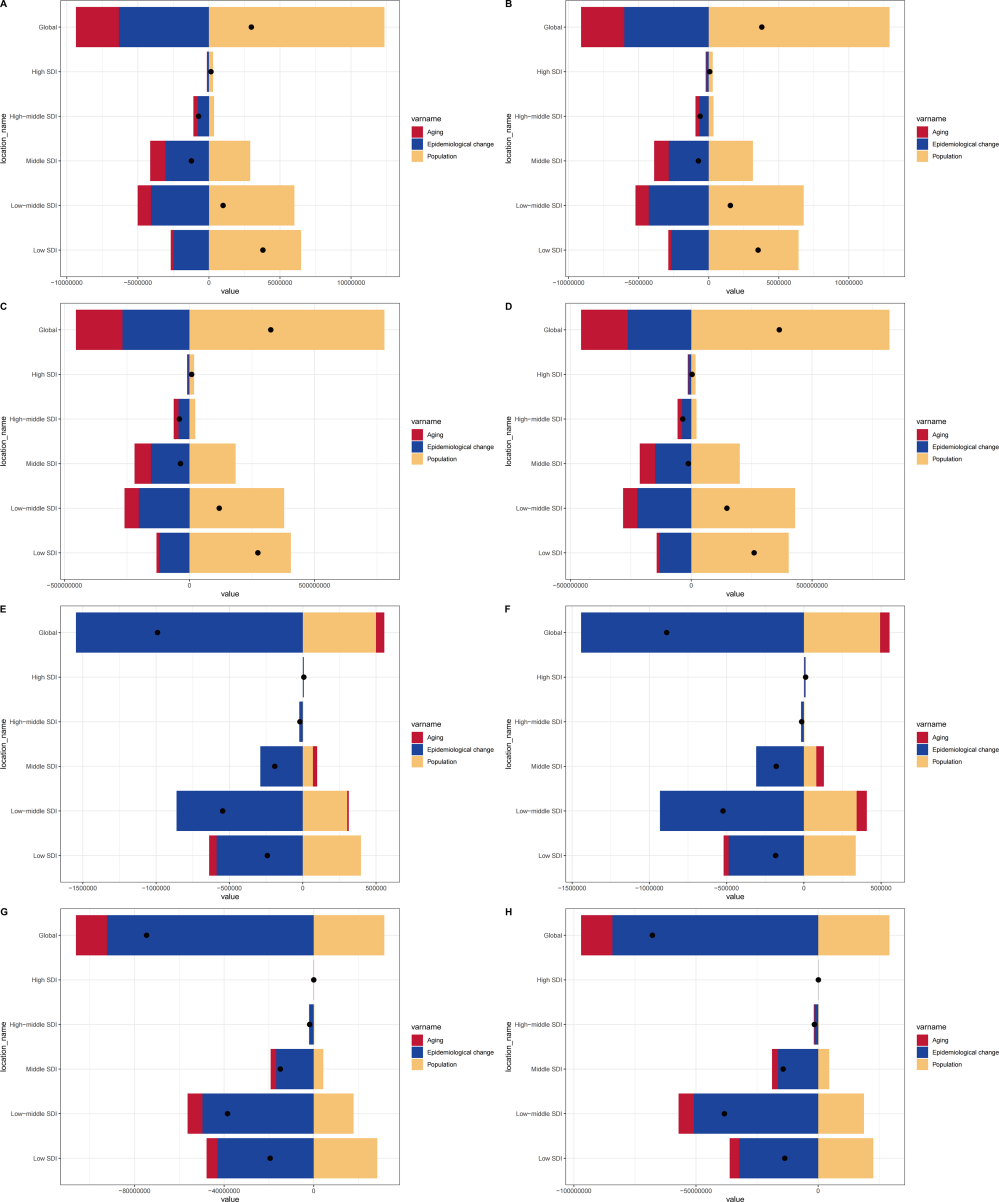
Changes in prevalence, incidence, deaths, and DALYs for both sexes according to population-level determinants of population growth, aging, and epidemiological change from 1990 to 2021 in the globe and SDI regions. **(A)** Changes in prevalence of males; **(B)** Changes in prevalence of females; **(C)** Changes in incidence of males; **(D)** Changes in incidence of females; **(E)** Changes in deaths of males; **(F)** Changes in deaths of females; **(G)** Changes in DALYs of males; **(H)** Changes in DALYs of females. SDI, Socio-demographic index; DALYs, Disability-adjusted life years.

From 1990 to 2021, the increase in prevalence and incidence globally and in High SDI, Low-middle SDI, and Low SDI regions was primarily due to population growth. In High-middle SDI and Middle SDI regions, there was a decrease, mainly due to epidemiological changes. The reduction in deaths and DALYs across all four SDI regions (except High SDI) was primarily due to epidemiological changes. In High SDI regions, the increase was attributed to aging for deaths in males (45.62%) and epidemiological changes for deaths in females (44.17%). The increase in DALYs was attributed to population growth (**Figure 6**; **Supplementary Table S59**).

## Discussion

Enteric infections represent a significant public health challenge worldwide, with a substantial disease burden that has attracted considerable research and attention. This study comprehensively estimated the prevalence, incidence, deaths, and DALYs of enteric infections across 21 global regions, 204 countries and territories, and age groups, as well as risk factors, providing a detailed picture of the disease burden of enteric infections. The main findings are as follows:

### Regional variability and risk factors

High-ranking regions for ASPR and ASIR are Low SDI and South Asia, while ASMR and ASR of DALYs are highest in Low SDI and Western Sub-Saharan Africa. America than in South Asia and in sub-Saharan Africa, but higher than in high-income regions. Unsafe water sources contribute significantly to high DALYs and deaths in Low SDI regions, accounting for up to 65.4% and 63.8% respectively. Limited water infrastructure and the use of contaminated drinking water are significant factors (Bain et al., 2014). Inadequate water facilities increase exposure to contaminated sources. In rural Bangladesh, the risk of neonatal death is 3.12 times higher in households without toilets than in those with toilets (Rahman et al., 1985). In many developing areas, untreated water from wells and rivers is used for drinking, bathing, and irrigation (Laffite et al., 2016), leading to severe fecal contamination, especially in rainy and dry seasons (Kayembe et al., 2018).In Latin America and the Caribbean, as well as in East Asia and the Pacific region, the reduction in temperature-attributable enteric infection deaths caused by viral pathogens has led to a net decrease in overall temperature-related enteric infection mortality (Verlicchi et al., 2010).

ASMR and ASR of DALYs decreased with increasing SDI, but the regions with the fastest growth in ASMR and the slowest decrease in ASR of DALYs were High SDI regions, specifically High-income North America and Western Europe. In these high-income areas, policies and facilities have reduced the impact of unsafe water resources on enteric infection-related diseases, but hospital wastewater may become a new source of infection (Boillot et al., 2008; Verlicchi et al., 2010), with its complex mixture of chemicals and biological substances potentially promoting the spread of bacteria and their antibiotic resistance genes (Cosgrove, 2006).

From 1990 to 2021, the global and 21 GBD regions showed a downward trend in ASPR and ASIR with increasing SDI, reflecting the positive impact of socio-economic development on health status improvement. Notably, in 2021, High-income Asia Pacific ranked high in ASPR and ASIR, which, in addition to unsafe water resources, may also reflect the high sensitivity or high-risk exposure of specific populations in the region, such as the elderly, chronic disease patients, or immigrant groups, to enteric infections. The systemic inflammation that occurs during aging can lead to dysbiosis (Li et al., 2016), and the microbiota that was dominant in youth can become pathogen-rich, such as Enterobacteriaceae (Fransen et al., 2017), increasing the risk of disease. The high-fat and high-sugar dietary habits common in High-income Asia Pacific may promote dysbiosis (Randeni et al., 2024). Also, physical activity, stress levels, sleep patterns, medication use, and even mental state can affect the gut microbiome (Mundula et al., 2022; Mundula et al., 2023). Over the past three decades, environmental issues and international travel have significantly increased transmission risk and infection prevalence of enteric diseases (Love et al., 2024). So the incidence of disease increases significantly for those who have traveled to South Asian countries such as India, Nepal, and Bhutan (Kuenzli et al., 2017).

### Subtype analysis

In 2021, typhoid fever was the leading cause of high intestinal infections (prevalence and incidence) in Central Latin America and Southeast Asia (**Supplementary Figure S1**). A systematic review and meta-analysis of case-control studies assessing the association between typhoid and WASH as well as food exposure suggested that poor sanitation and untreated water had the strongest correlation with typhoid risk (Brockett et al., 2020). Moreover, warm and humid climates likely contribute to pathogen survival and growth, with higher temperatures enhancing their proliferation (Lal et al., 2012). Furthermore, Salmonella typhi has developed strong drug resistance (Browne et al., 2020). From a low base in 1990, the prevalence of fluoroquinolone-resistant Salmonella typhi surged, reaching an overall prevalence of 95.2% in South Asia by 2019 (Zakir et al., 2021; GRAM Typhoid Collaborators for 2024, 2024). Since 2016, Pakistan has experienced an outbreak of typhoid caused by extensively drug-resistant (XDR) S., which may be closely related to international air travel (Walker et al., 2023).

It is noteworthy that in High-income North America and High-income Asia Pacific regions, typhoid fever accounts for the smallest proportion of enteric infection deaths and DALYs; instead, iNTS has become the main cause of death, mainly related to the difficulty in treating iNTS and its multiple complications (**Supplementary Figure S1**). A meta-analysis indicated that a significant proportion of patients with non-typhoidal Salmonella invasive diseases have life-threatening complications such as encephalopathy and pleuropulmonary infections. Among the 84 studies it summarized, 66 (78.6%) had a high overall bias risk, 18 (21.4%) were at moderate risk, none were at low risk, and about 15% of patients with non-typhoidal Salmonella invasive diseases eventually died (Marchello et al., 2022), highlighting the deadliness of iNTS.

### Age and gender analysis

Age and gender both influence the gut microbiota (Singh and Manning, 2016). According to age analysis, the burden is heavier on infants, adolescents, and the elderly (**Figure 2**). The richness of the microbial community increases with age in children, but it is still lower than the diversity of the adult microbiota at the age of 5 (Roswall et al., 2021). Additionally, the mode of birth, microbiota-directed food, and other factors may be related to the gut microbiota in early life (Gehrig et al., 2019). Furthermore, whether mothers wash their hands before breastfeeding and after cleaning the child, the child’s use of toilet facilities and the child’s hygiene habits such as washing hands before meals and not putting hands in the mouth can also be contributing factors.

Elderly individuals often acquire frailness-associated bacteria like low Bifidobacterium (Pang et al., 2023). Long-term medication use can lead to abnormal microbiomes and metabolomes in the elderly (Forslund et al., 2021; Li et al., 2024), contributing to higher death rates, along with physical decline and hospital-acquired infections (Taylor and Oppenheim, 1998). Malnutrition, including child wasting, stunting, underweight, and unsafe sanitation significantly increase the risk of intestinal infections in children, with 48.7% of malnourished children in southern Ethiopia affected by parasitic infections (Yoseph and Beyene, 2020).

The greater burden of prevalence and incidence in females than in males may be largely influenced by cultural customs. In rural communities in West Bengal, India, logistic regression analysis showed that boys had 4.2 times more opportunity to spend more money on medical treatment and were 4.9 times more likely to receive early medical care than girl, with the highest gender bias among mothers with higher education (Bhan et al., 2005).

### Etiological analysis

In the field of enteric infections, there have been significant changes in the distribution of causes and the resulting deaths and DALYs in recent years. From 1990 to 2021, Norovirus, Enterotoxigenic E. coli, and Cryptosporidium have continued to pose threats worldwide, but Rotavirus has always dominated, especially in causing deaths from intestinal infections in children.

However, it is noteworthy that over the past 32 years, there has been a significant decrease in the number of deaths and DALYs caused by various etiologies of enteric infections. Taking Rotavirus as an example, the global deaths it caused dropped from 392,147 in 1990 to 92,607 in 2021, and DALYs decreased from 32,857,936 to 7,279,772, mainly due to the widespread vaccination against Rotavirus and the strengthening of global healthcare (Soares-Weiser et al., 2019). Since 2006, vaccines such as RotaTeq, Rotarix, and Rotavac, which have been proven to significantly prevent Rotavirus infection have gradually been approved and launched (Wiedermann and Kollaritsch, 2006). Governments and research institutions around the world are also continuously exploring the development and application of new Rotavirus vaccines to achieve better prevention outcomes (ACIP recommends new vaccine to prevent rotavirus. An update from CDC, 2006).

### Overall and future trends analysis

From 1990 to 2021, global prevalence and incidence of enteric infections saw turning points in 2011 and 2015, likely due to increased vaccine R&D investments by WHO and governments, especially the broader use of vaccines like Rotavirus (Muhsen et al., 2015; Enane et al., 2016; Kobayashi et al., 2018).

In high SDI regions, the downward trend in prevalence and incidence rates for both males and females reversed to an upward trend in 2019 (Leung et al., 2003). The emergence of COVID-19 in 2019 was primarily a respiratory infection, often accompanied by gastrointestinal involvement, and up to half of patients’ gastrointestinal symptoms preceded respiratory symptoms, which complicated the treatment of intestinal infections (Jin et al., 2022). COVID-19 or antiviral treatments may also cause liver damage, adding to the challenge of managing enteric infections (Chen et al., 2020). Despite this, deaths and DALYs showed a downward trend across all SDI regions, except for high SDI. This could be attributed to advancements in medical technology and increased medical resources, significantly improving treatment outcomes for intestinal infectious diseases (Zhou et al., 2023; Cheng et al., 2024). Early detection, diagnosis, and treatment have also reduced the disease’s impact on patients, thereby reducing DALYs (Zhou et al., 2023; Cheng et al., 2024).

### Limitations

However, this study also has limitations. First, the GBD database primarily compiles data from national and regional reports and publications, rather than direct national reports, which may lead to issues with data completeness, timeliness, and quality, especially in low-income areas where there may be a lack of access to raw data, potentially hindering GBD researchers in their estimations. Additionally, differences in disease management across countries and regions, including diagnosis, recording, and reporting, may affect the results.

## Data Availability

The original contributions presented in the study are included in the article/**Supplementary Material**. Further inquiries can be directed to the corresponding authors.
