# Supplementary figures and images for: Global burden, subtype, risk factors and etiological analysis of enteric infections from 1990-2021: population based study
Source: Front Cell Infect Microbiol. 2025 Mar 20;15:1527765. doi: 10.3389/fcimb.2025.1527765 (PMC11965617; doi:10.3389/fcimb.2025.1527765)

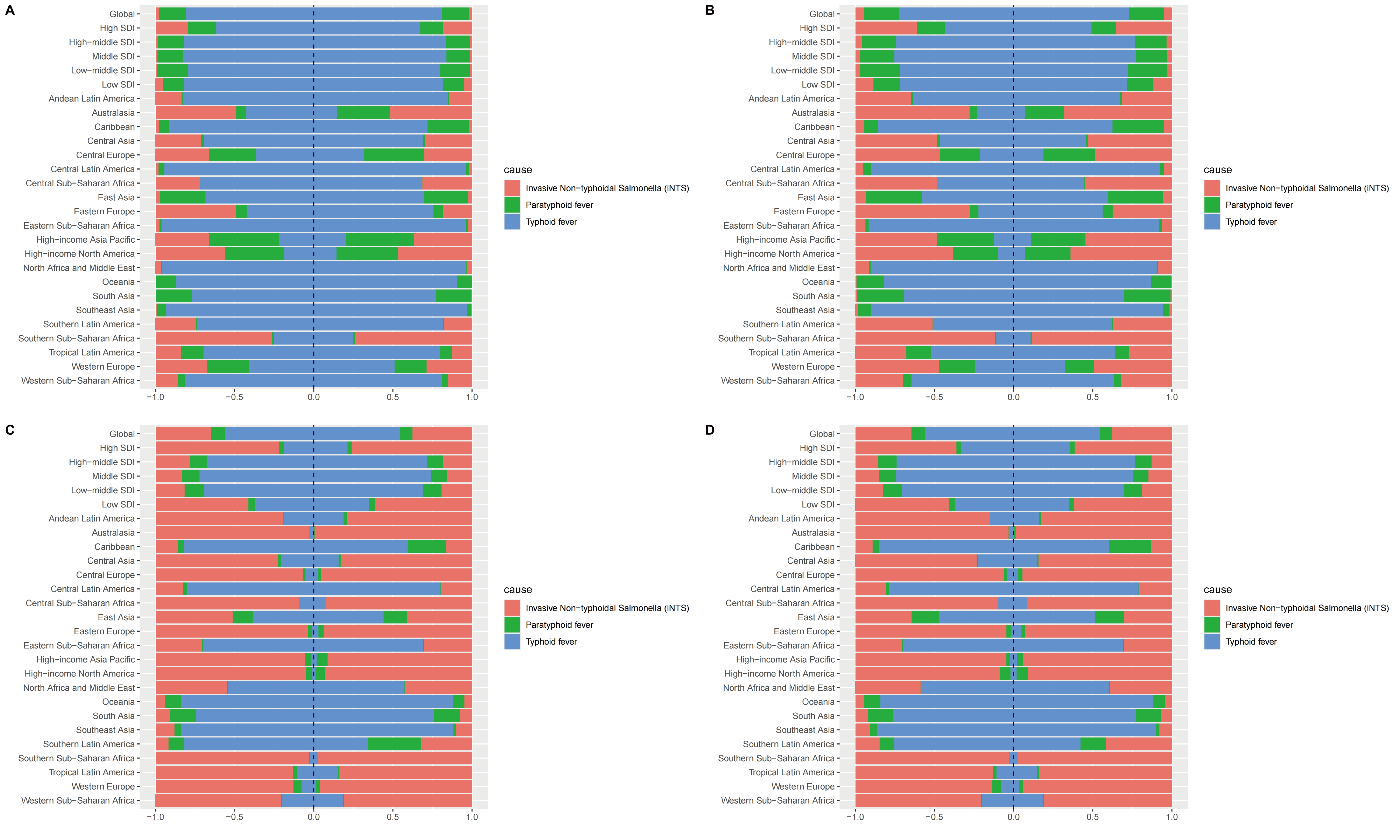

Supplement: Supplementary file 1 [file Image1.tif]

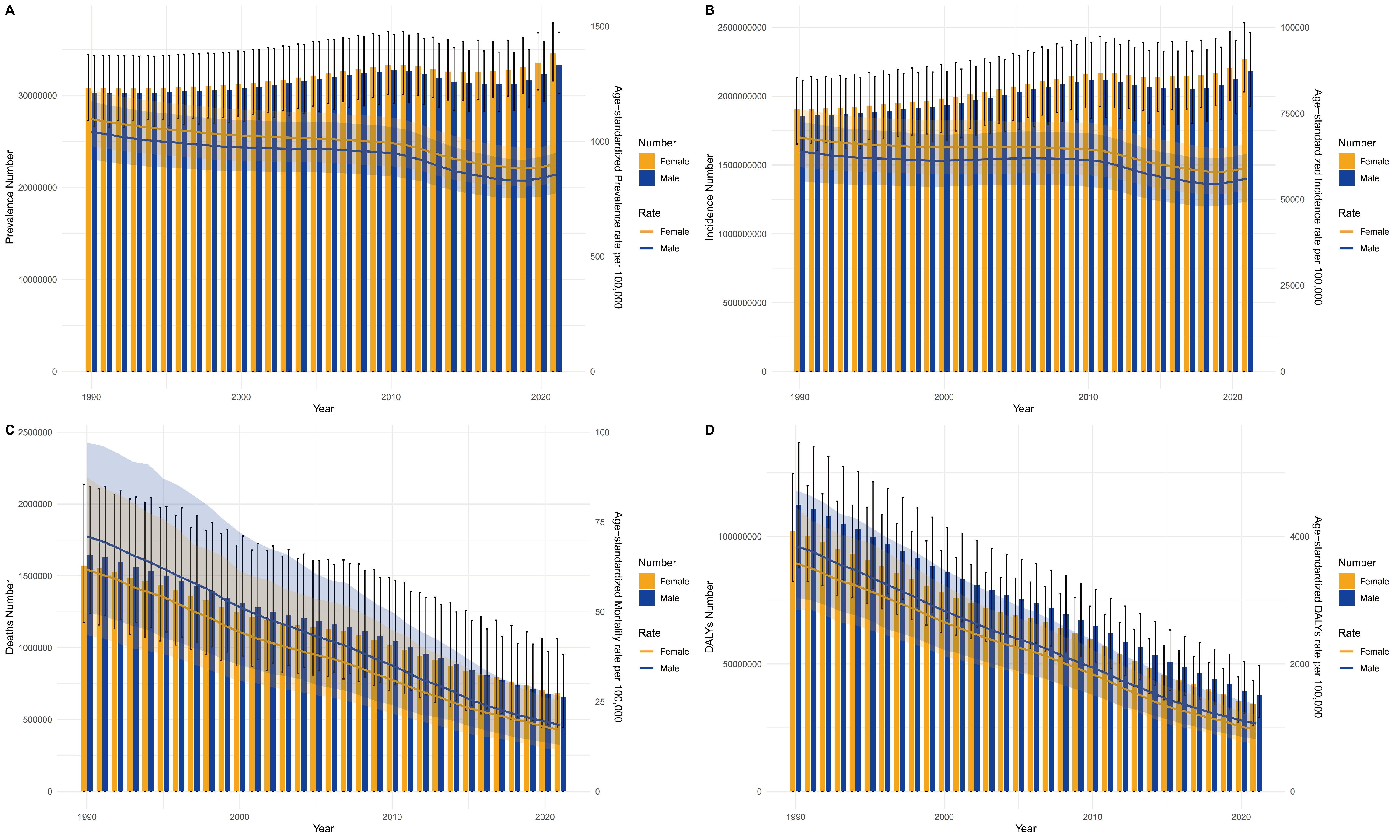

Supplement: Supplementary file 2 [file Image2.tif]

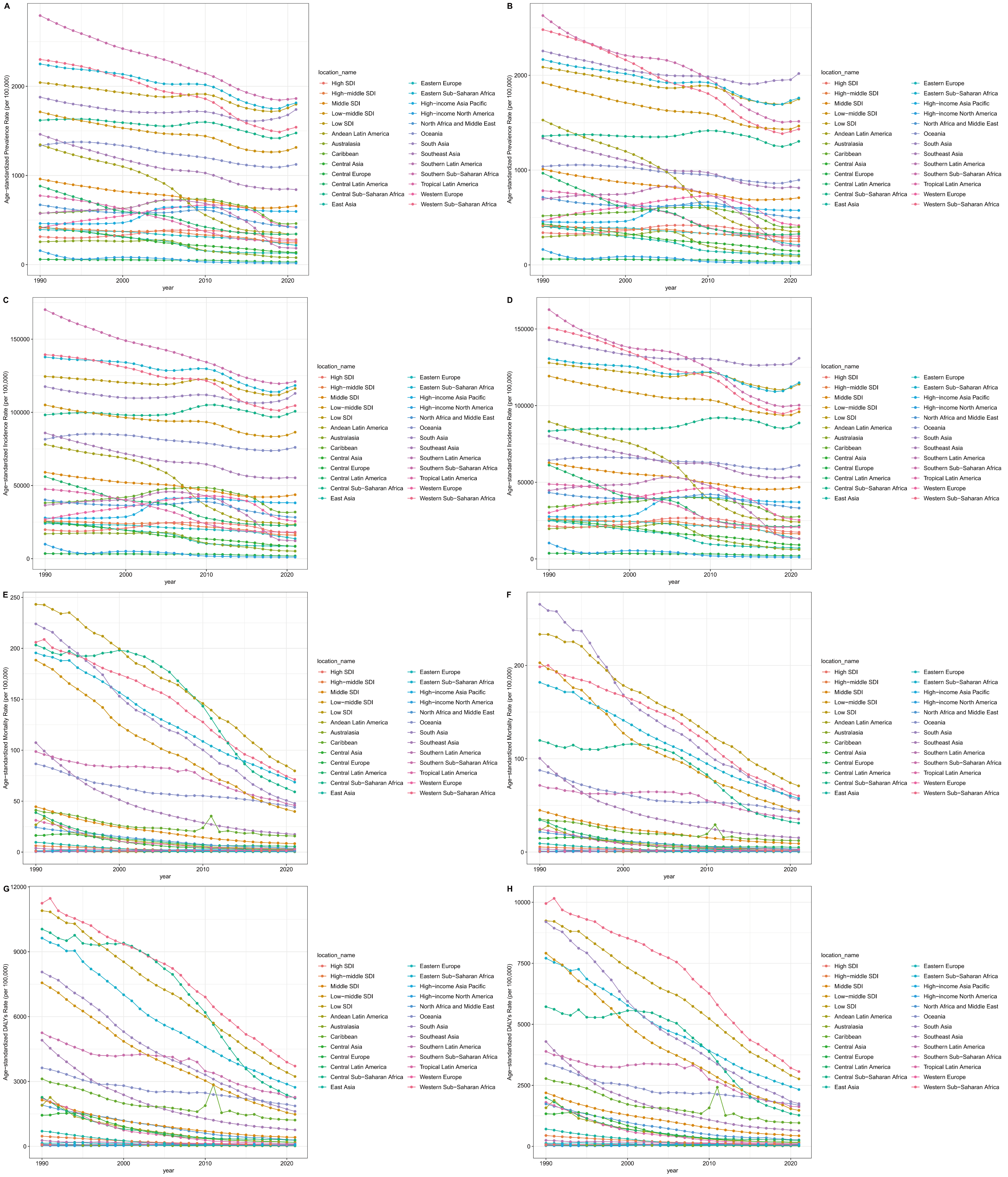

Supplement: Supplementary file 3 [file Image3.tif]

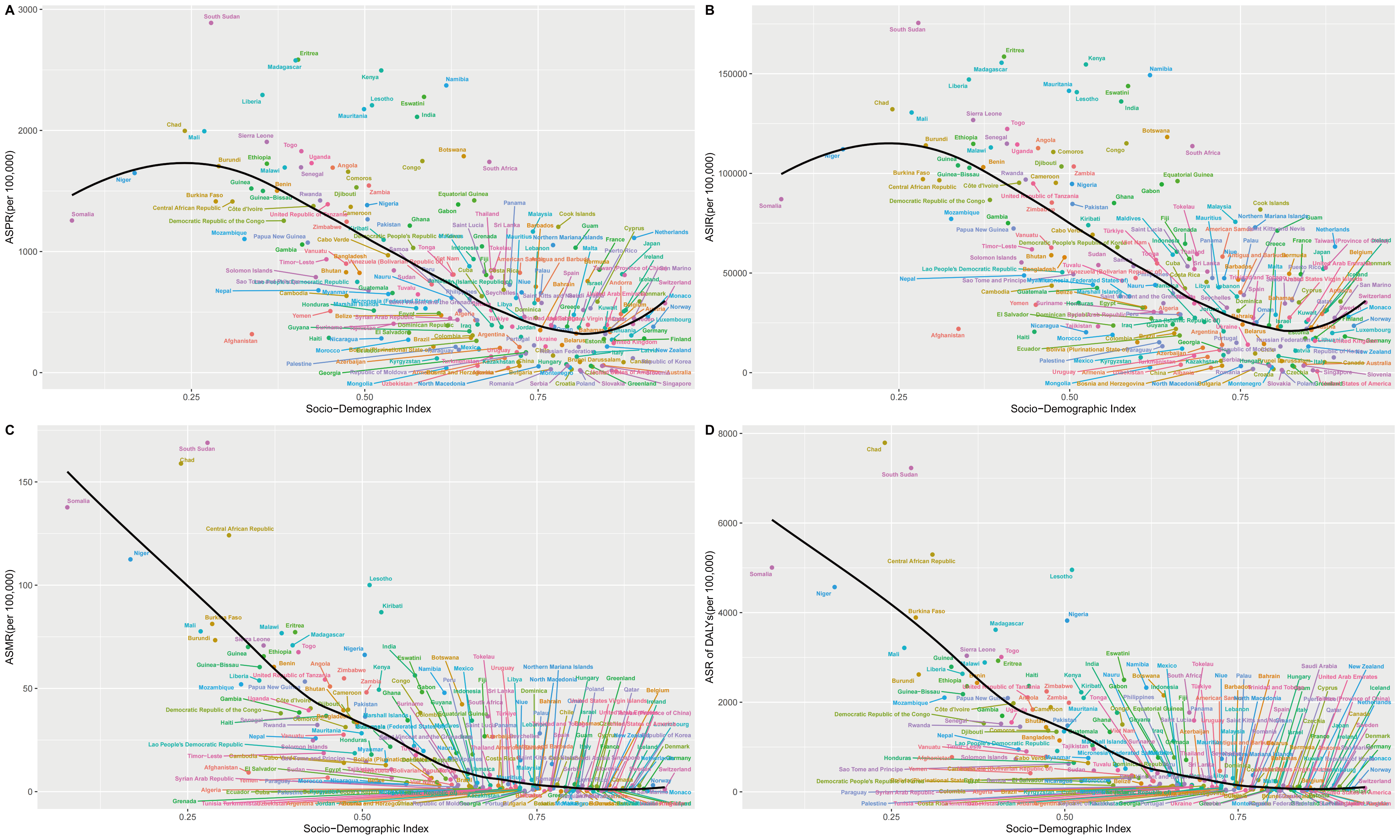

Supplement: Supplementary file 4 [file Image4.tif]

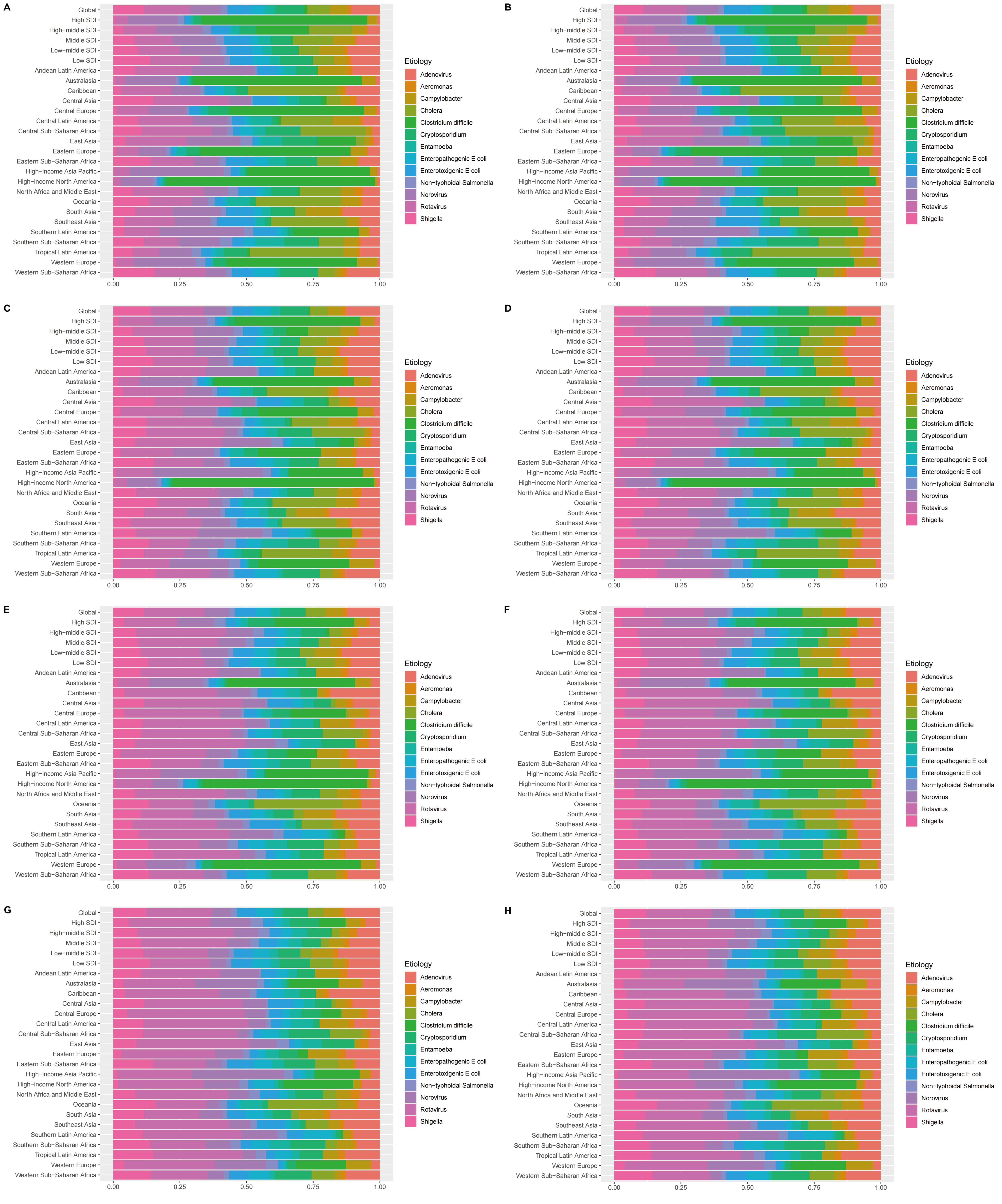

Supplement: Supplementary file 5 [file Image5.tif]
